# Comprehensive analysis of cuproptosis-related genes in immune infiltration and diagnosis in ulcerative colitis

**DOI:** 10.3389/fimmu.2022.1008146

**Published:** 2022-10-25

**Authors:** Jinke Huang, Jiaqi Zhang, Fengyun Wang, Beihua Zhang, Xudong Tang

**Affiliations:** ^1^ Department of Gastroenterology, Xiyuan Hospital of China Academy of Chinese Medical Sciences, Beijing, China; ^2^ Institute of Digestive Diseases, Xiyuan Hospital of China Academy of Chinese Medical Sciences, Beijing, China

**Keywords:** Ulcerative colitis, cuproptosis, genes, immune infiltration, diagnosis

## Abstract

**Objectives:**

Cuproptosis is a recently discovered form of programmed cell death; however, its role in ulcerative colitis (UC) remains a void.

**Methods:**

Three gene expression profiles were acquired from the GEO database. Subsequently, the single sample gene set enrichment analysis (ssGSEA) was performed to identify the immune infiltration characteristics of UC. Correlation analysis between cuproptosis and immune infiltration was further conducted, and the cuproptosis-related genes were applied to construct a UC diagnostic model. Subsequently, analysis results of microarray data were experimentally validated by DSS-induced colitis in mice. Finally, therapeutic agents for the cuproptosis-related genes were screened owing to the gaping field of therapeutic agents on cuproptosis.

**Results:**

Three gene expression profiles with 343 samples (290 UC and 53 healthy samples) were included. Immune infiltration revealed that UC patients had a higher level of DCs, B cells, CD8^+^ T cells, iDCs, Macrophages, neutrophils, pDCs, T helper cells, Tfh, Th1 cells, Th2 cells, TIL and Treg than normal subjects. Moreover, almost all cuproptosis-related genes were significantly negatively associated with immune infiltration in UC patients. The risk prediction model based on cuproptosis-related genes showed an excellent discrimination for UC. Animal experiments revealed significant alterations in genes essential for cuproptosis between DSS-induced colitis mice and healthy controls, providing experimental validation for the analysis results of microarray data. Further analysis revealed that latamoxef, vitinoin, clomipramine, chlorzoxazone, glibenclamide, pyruvic acid, clindamycin, medrysone, caspan, and flavin adenine dinucleotide might be the target agents for cuproptosis-related genes.

**Conclusions:**

In conclusion, cuproptosis was significantly associated with immune infiltration in UC, and the cuproptosis-related genes showed an excellent discrimination for UC.

## 1 Introduction

Ulcerative colitis (UC) is a complex disease characterized by chronic inflammation of the colon (1). Worldwide, UC is estimated to affect 9-12/100,000 people annually, and the incidence is increasing year by year (2). The growing number of UC patients places a heavy economic burden on society, with direct and indirect costs associated with UC of $8.1 – $14.9 billion per year in the United States and €12.5 – 29.1 billion in Europe (3). The treatment goal in UC is the induction and maintenance of remission. Although therapeutic tools are expanding, the treatment of UC is highly challenging because of its incompletely understood pathogenesis (4). Therefore, an in-depth understanding of disease pathogenesis and identification of biomarkers of disease progression at the molecular level may provide new ideas for the early diagnosis of UC.

**Figure 1:**
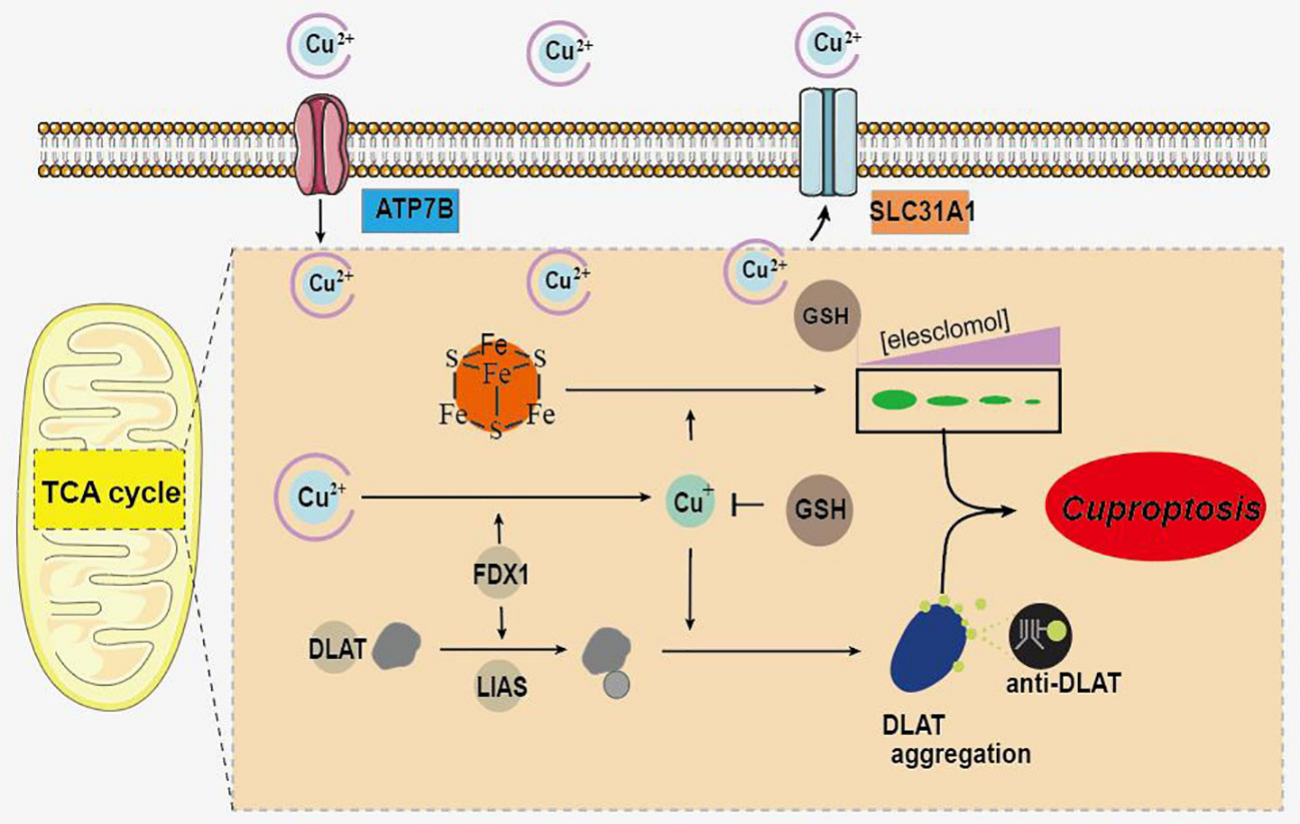
Schematic of mechanisms promoting cuproptosis.

It is reported that various types of cell death are closely related to UC, and the regulation of cell death is an important strategy for its treatment (5). Recently, a novel copper-dependent cell death mediated by proteolipid acylation has been identified and termed “cuproptosis” (6). As a metal trace, copper is involved in a variety of physiological activities in living organisms and is essential for the maintenance of normal biological activities (7, 8). Copper deficiency impairs the function of copper-binding enzymes, and cell death can be induced by excess copper (9). Excess intracellular copper has been reported to bind directly to the lipid acylated components of the tricarboxylic acid cycle, leading to lipoylated protein aggregation and subsequent iron-sulfur cluster protein loss (6). This process leads to proteotoxic stress and ultimately to cuproptosis (**Figure 1**) (6). In addition, FDX1 and protein lipoylation are identified as the key regulators of copper ionophore induced cell death (6).

**Figure 2:**
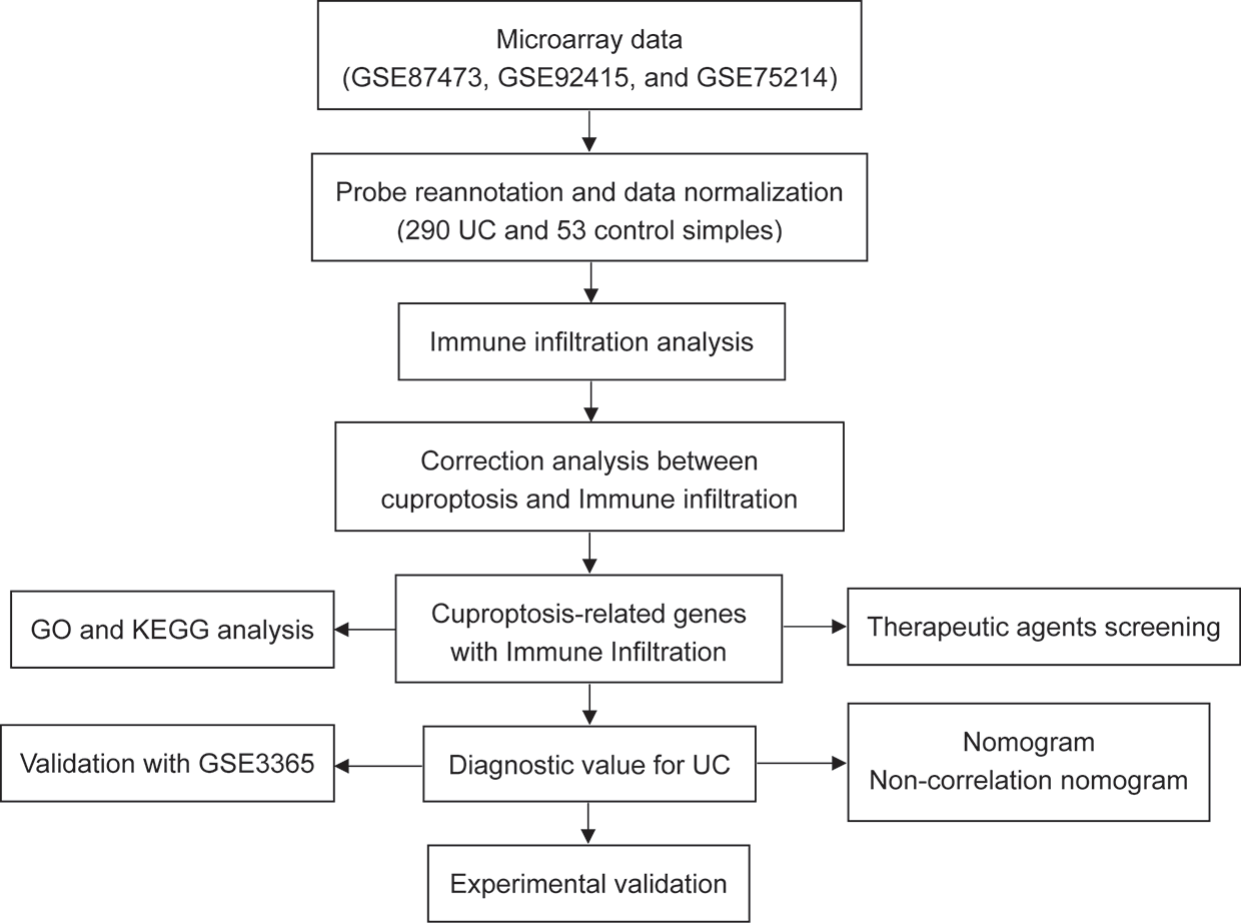
Flowchart of the present research.

Accumulating evidence revealed that significant abnormalities in copper metabolism were present in UC patients and were strongly associated with its development (10–13). Furthermore, copper-containing metabolic structural domain 1 suppresses genes that promote inflammation and protects mice from colitis and colitis-associated cancers (14). Similarly, copper-mediated oxidation of mesalazine, a pro-oxidant interaction through a copper redox cycle mechanism, may exert anti-cancer effects in patients with ulcerative colitis (15). Moreover, cumulative evidence indicated that copper has also been involved in the regulation of the immune system (16–18). All these findings suggest an important role of copper in UC. However, as a newly identified form of regulated cell death, the role of cuproptosis in the pathogenesis, development, and immune system of UC remains a void, and its potential in being the therapeutic target for UC is far to be understood. Therefore, we hypothesized that cuproptosis was involved in UC and that cuproptosis-related genes may contribute to the early diagnosis and treatment of UC. The workflow of the present study is shown in **Figure 2**.

## 2 Materials and methods

### 2.1 Microarray data acquisition

Gene expression profiles were acquired from GEO database (www.ncbi.nlm.nih.gov/geo/) with the following criteria: (a) patients were diagnosed as UC; (b) data on colonic tissue from healthy controls and UC patients from the same GEO platform; (c) inclusion of datasets with at least 10 UC and healthy tissue samples; (d)) GEO platforms containing >5000 genes. Finally, three gene expression profiles (GSE87473, GSE92415, and GSE 75214) were included (**Table 1**). The batch effects among these datasets were eliminated by applying the combat algorithm in the “sva” R package (https://www.bioconductor.org/packages/release/bioc/html/sva.html). The “sva” package can remove batch effects in three ways: (1) identifying and estimating surrogate variables for unknown sources of variation in high-throughput experiments (19), (2) removing known batch effects directly using ComBat (20) and (3) removing batch effects with known control probes (21). Removal of batch effects and use of surrogate variables in differential expression analysis has been shown to reduce dependence, stabilize error rate estimates, and improve reproducibility.

**Table 1:** Details of microarray data.

Dataset	Platform	Tissue	UC	Normal	Reference (PMID)
GSE87473	GPL13158	Colon	106	21	29401083
GSE75214	GPL6244	Colon	97	11	28885228
GSE92415	GPL13158	Colon	87	21	23735746

Furthermore, cuproptosis-related genes (FDX1, LIPT1, LIAS, DLD, DBT, GCSH, DLST, DLAT, SLC31A1, PDHB, PDHA1, ATP7A, and ATP7B) were obtained from previous literature (16).

### 2.2 Immune infiltration analysis

Single sample gene set enrichment analysis (ssGSEA) was performed to define the immune infiltration status between control and UC samples by calculating the normalized enrichment score. *P* < 0.05 was used to filter the samples. Heat map of immune infiltration in samples was produced by the “pheatmap” package (https://cran.r-project.org/web/packages/pheatmap/). Levels of immune cells and immune function between UC and control samples were visualized by the “vioplot” package (https://cran.r-project.org/web/packages/vioplot/).

### 2.3 Correlation analysis between immune infiltration and cuproptosis-related genes

The correlation between the cuproptosis-related genes and immune infiltration in UC was evaluated by the “psych” (https://cran.r-project.org/web/packages/psych/) and “ggcorrplot” (https://cran.r-project.org/web/packages/ggcorrplot/) packages. The “ggplot2” package (https://sourceforge.net/projects/ggplot2.mirror/) was performed to analyze results and *p* < 0.05 was considered significant.

### 2.4.The construction of risk prediction model

After the feature selection, the cuproptosis-related genes most strongly associated with immune infiltration were used to construct risk prediction model for UC with “rms” package (https://cran.r-project.org/web/packages/rms/). The prediction performance of the model was quantified by nomogram, non-correlated nomogram, and ROC curve. ROC-AUC ≥ 0.9 indicates outstanding discrimination. 0.8≤ ROC-AUC < 0.9 indicates excellent discrimination, 0.7 ≤ ROC-AUC < 0.8 indicates acceptable discrimination, and ROC = 0.5 indicates no discrimination (22). ROC analysis was performed with “ROCR” package (https://www.rdocumentation.org/packages/ROCR/).

### 2.5 Enrichment analysis for cuproptosis-related key genes

Enrichment analysis of GO and KEGG were performed for the cuproptosis-related genes in our model by the Enrichr (https://maayanlab.cloud/Enrichr/). Results of enrichment analysis were visualized with the “ggplot2” package (https://sourceforge.net/projects/ggplot2.mirror/).

### 2.6 Therapeutic agents screening for cuproptosis-related key genes

Therapeutic agents for the cuproptosis-related key genes were screened using Enrichr (https://maayanlab.cloud/Enrichr/). The threshold for enrichment analysis was set to *p*-value < 0.05.

### 2.7 Experimental validation

Twelve male C57BL/6J mice (18–22 g) were obtained from SPF Biotechnology Co., Ltd (Beijing, China). Before starting the experiment, animals were fed with free access to food and water for seven days to adapt to the environment. Animal experimental protocols were performed with the approval of the Animal Ethics Committee of Xi Yuan Hospital of China Academy of Chinese Medical Sciences (Approval NO. 2019XLC003-2).

Animals were randomly divided into control group and dextran sulfate sodium (DSS) group. To induce acute experimental colitis, mice in the DSS group were given 3.0% (w/v) DSS (Cat. No. 160110, MP Biomedicals) in the drinking water ad libitum for 7 days. Control group received regular diet and drinking water throughout the experimental period. Body weight, stool consistency, and rectal bleeding of all animals were recorded daily. At the end of the experiment, mice were sacrificed, the colonic tissues were quickly excised and measured for length, and then stored in the refrigerator at -80°C until use (**Figure 8A**).

#### 2.7.1 Histological analysis

Colon tissue was fixed in 4% paraformaldehyde and then processed into paraffin-embedded tissue blocks to produce 5 µm-thick sections for hematoxylin and eosin (H&E) staining, in which a blinded colitis activity score, according to previous criteria (23), was given.

#### 2.7.2 Immunohistochemistry

Immunohistochemistry (IHC) was performed with paraffin‐embedded sections. Slices were incubated with ZO-1 (Cat. No. AF5145, Affinity Biosciences, 1:100), occludin (Cat. No. 27260-1-AP, Proteintech, 1:200), claudin-1 (Cat. No. 28674-1-AP, Proteintech, 1:200), FDX1 (Cat. No. BS-11426R, Bioss, 1:400), LIAS (Cat. No. 11577-1-AP, Proteintech, 1:200) and DLAT (catalog no. 13426-1-AP, Proteintech, 1:300) antibody overnight at 4°C. Later, the slides used for IHC staining were incubated with the secondary antibody (Cat. No. GB23303, Servicebio, 1:200). The DAB chromogen was used for incubation, and then hematoxylin was used for counterstaining. Finally, images were acquired under a light microscope (Olympus BX41, Shanghai, China). Staining intensity was analyzed using Image Pro Plus 6.0 (Media Cybernetics, Inc., Rockville, MD, USA).

#### 2.7.3 Quantitative real-time PCR

Total RNA was extracted from colon tissue and reverse transcribed as cDNA template. Next, quantitative real-time PCR (qRT-PCR) was carried out with the CFX96 real-time PCR detection system (Bio-Rad, USA). The expression levels of target genes were analyzed with 2^-ΔΔCT^, and the results were presented with GAPDH as an internal control. Primer sequences in this study are listed in **Table 2**.

**Table 2:** The primer sequences were used in this study.

Genes	Forward primer	Reverse primer
FDX1	ACAGACAGGAACCTGGAAGACC	GAGACAATCTGTATGGGGTGGTT
LIAS	CGTTAAGACCGCAAGAAATCC	CCACATCATCTCGATCCACC
DLAT	TCACAGACATCCCCATCAGCA	TTAAGTTCCTTCCGTACCAACAG
GAPDH	CCTCGTCCCGTAGACAAAATG	TGAGGTCAATGAAGGGGTCGT

### 2.8 Statistical analysis

Categorical variables are presented as percentages, while continuous variables are presented as the mean ± standard deviation. For bioinformatics analysis, the R software (Version 4.1.2, https://www.r-project.org/) was used for all data analysis in the present study. For experimental validation, student’s t-test was applied for comparisons between two groups with Prism GraphPad software (Version 7.04, https://www.graphpad.com/scientific-software/prism/). A value of *p* < 0.05 indicates statistically significant difference.

## 3 Results

### 3.1 Immune infiltration analysis for UC

**Figure 3:**
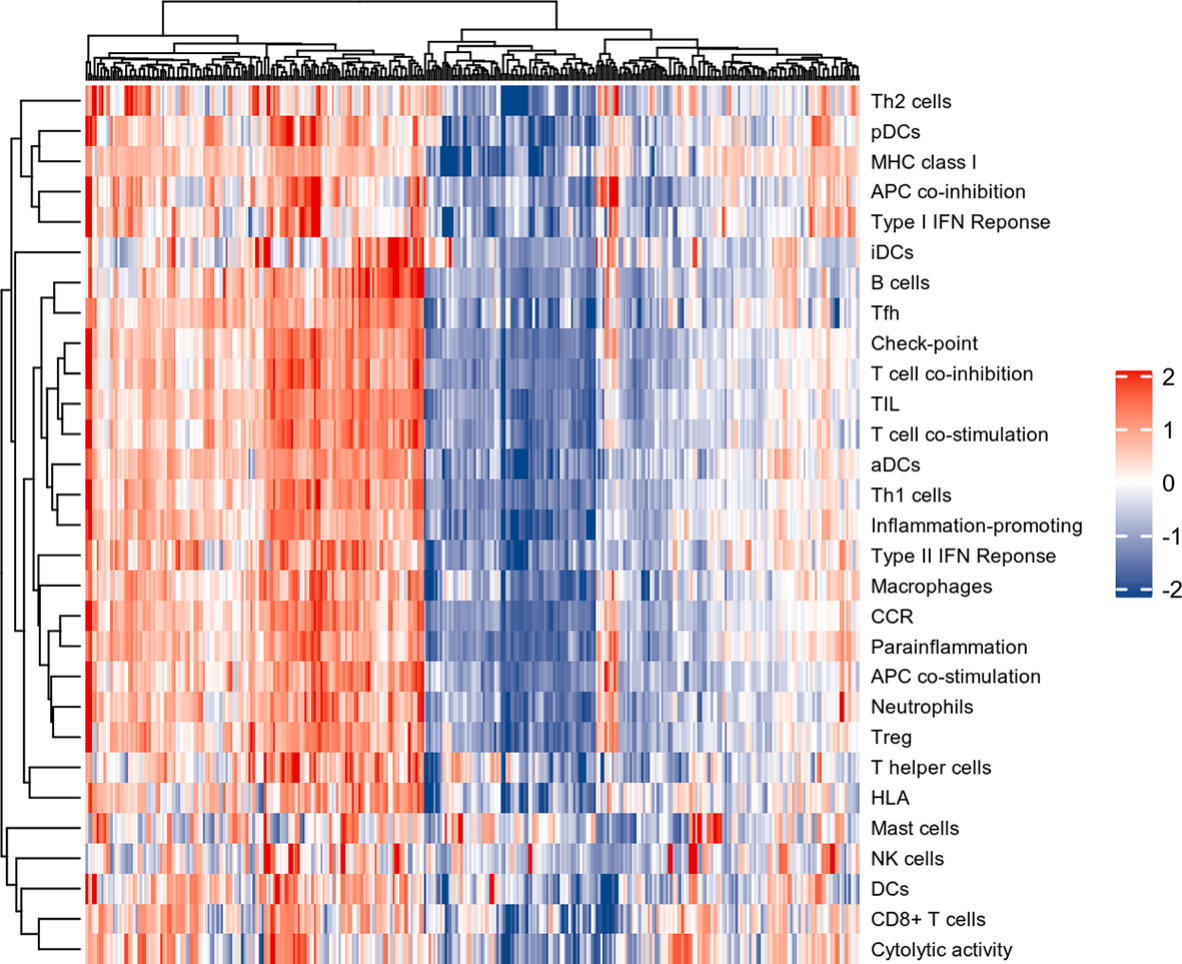
The heat map of Immune infiltration. Each row represents one sample, either normal or UC, each column represents a type of immune cell or immune function.

**Figure 4:**
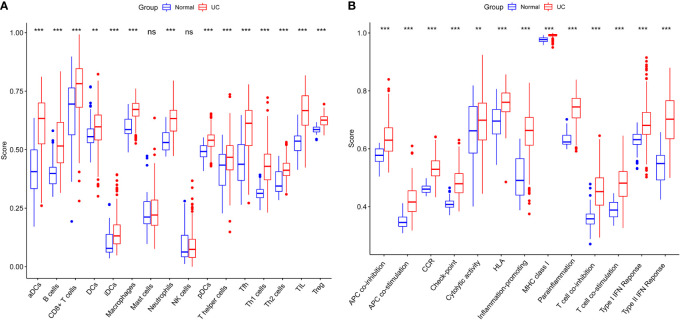
**(A)** Comparison of 16 immune cell subtypes between patients with UC and controls. **(B)** Comparison of 13 immune function subtypes between patients with UC and controls. *p < 0.05 compared to the normal, **p < 0.01 compared to the normal, ***p < 0.001 compared to the normal, ^ns^p > 0.05 compared to the normal.

The normalized enrichment score of immune infiltrates is presented in the heat map (**Figure 3**). The results of differential analysis of immune cell infiltration revealed that UC patients had a higher level of aDCs, B cells, CD8^+^ T cells, DCs, iDCs, macrophages, neutrophils, pDCs, T helper cells, Tfh, Th1 cells, Th2 cells, TIL and Treg than healthy subjects (**Figure 4A**). Moreover, a significant higher level of all immune function subtypes was observed in UC patients (**Figure 4B**).

### 3.2 Correlation analysis between immune infiltration and cuproptosis-related genes

**Figure 5:**
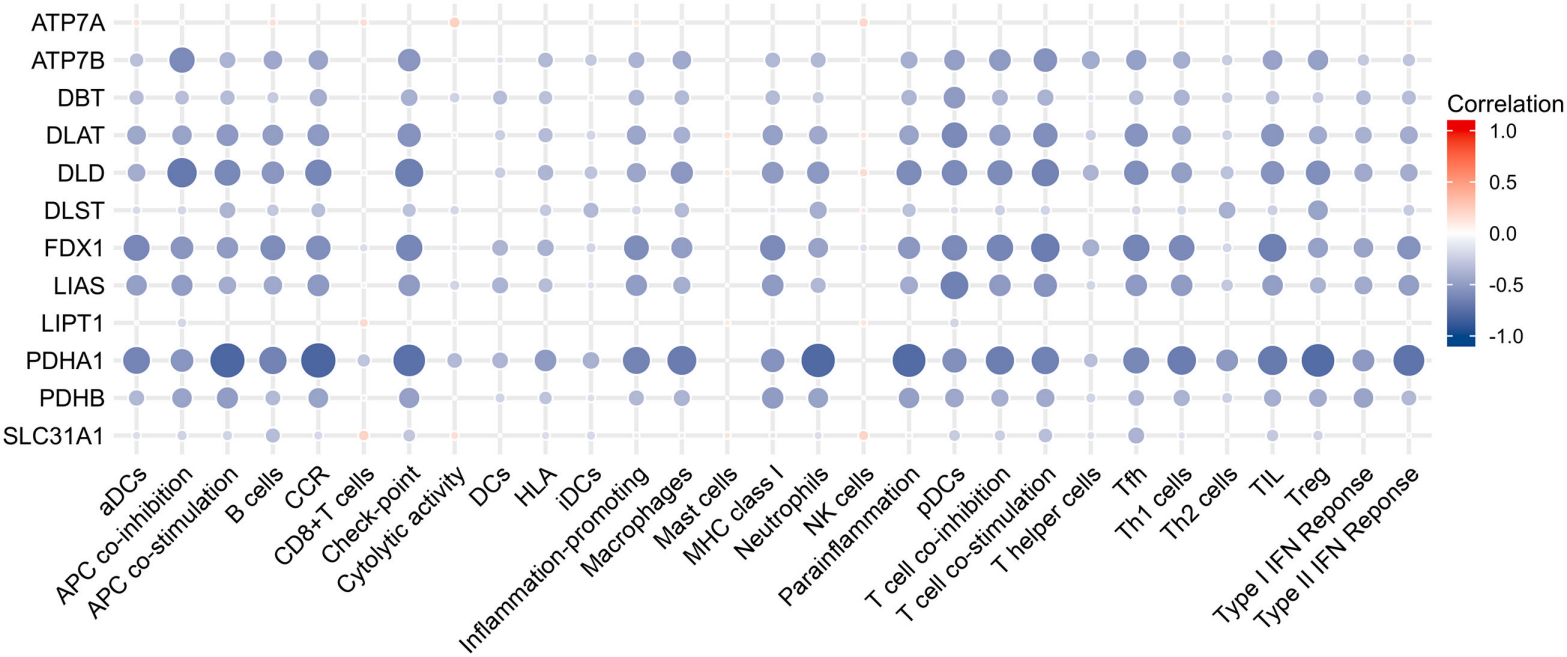
Correlation analysis between immune infiltration and cuproptosis-related genes.

Results of Pearson correlation analysis revealed that all cuproptosis-related genes were significantly negatively associated with almost all immune cell subtypes and immune function subtypes (except mast cells). PDHB, PDHA1, LIAS, FDX1, DLD, DLAT, DBT, and ATP7B were negatively associated with more than 24 immune cell subpopulations and immune function subtypes (**Figure 5**).

### 3.3 Construction of risk prediction model

**Figure 6:**
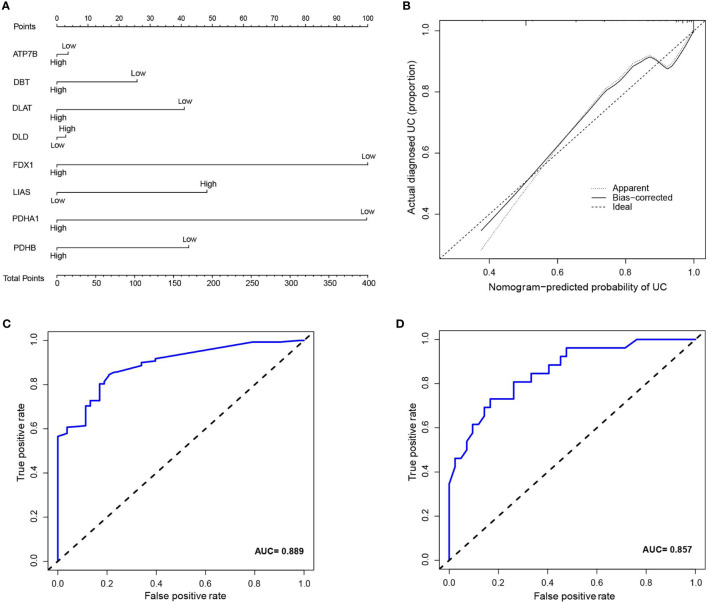
**(A)** nomogram of diagnostic marker genes. **(B)** Calibration curve of non-correlation nomogram prediction in the cohort. **(C)** ROC curve for the diagnostic efficacy of diagnostic model. **(D)** ROC curves analysis of test set in GSE3365.

Inspired by previous results, PDHB, PDHA1, LIAS, FDX1, DLD, DLAT, DBT, and ATP7B were used to construct a risk prediction model for UC. Coefficients of the cuproptosis-related genes in this model are presented in **Table 3**. The clinical information and genetic characteristics of UC patients were integrated to develop nomogram. Results showed that the constructed model with these predicted diagnostic genes had diagnostic efficacy for UC (**Figure 6A**). Based on the calibration curve predicted by the uncorrelated nomogram, the performance of the column line plot was close to the ideal model, suggesting that the predictive value of the model is reliable (**Figure 6B**). Similarly, ROC-AUC of the risk score was 0.889, that is an indicative of excellent model discrimination (**Figure 6C**). Furthermore, the microarray data of GSE3365 68 clinical simples (26 UC patients and 42 healthy controls) were used to verify the robustness of the model. Results revealed that ROC-AUC of the risk score was 0.857, indicating excellent model discrimination (**Figure 6D**).

**Table 3:** Coefficients of the cuproptosis-related genes in modle.

Genes	Estimate	Std. Error	z value	Pr (>|z|)
ATP7B	-0.08443	0.45697	-0.185	0.853423
DBT	-0.60574	0.41556	-1.458	0.144944
DLAT	-0.96232	0.50883	-1.891	0.058593
DLDH	0.06694	0.53848	0.124	0.901073
FDX1	-2.34911	0.64954	-3.617	0.000298
LIAS	1.13356	0.51425	2.204	0.027505
PDHA1	-2.34070	0.61410	-3.812	0.000138
PDHB	-0.99644	0.46688	-2.134	0.032823

### 3.4 Enrichment analysis for cuproptosis-related key genes

**Figure 7:**
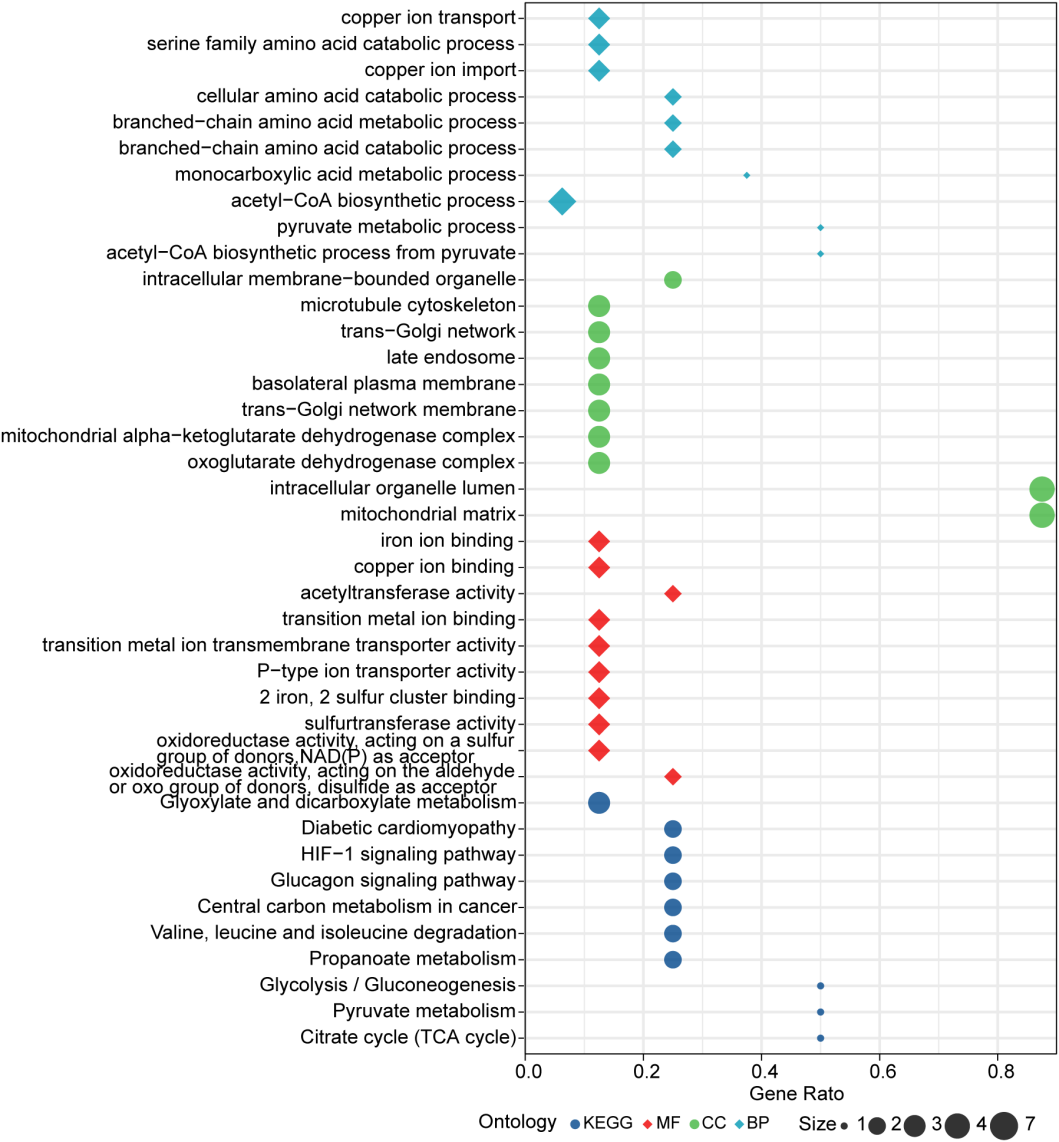
Results of GO and KEGG enrichment analysis.

Further, enrichment analysis for cuproptosis-related key genes in this model was performed. GO analysis revealed that these genes were significantly enriched in many metabolic pathways, such as: acetyl-CoA, pyruvate, monocarboxylic acid, branched-chain amino acid, cellular amino acid, copper ion transport and serine amino acid family (**Figure 7**). For KEGG analysis, genes were significantly enriched in citrate cycle (TCA cycle), HIF-1 signaling pathway, pyruvate metabolism, glycolysis/gluconeogenesis propanoate metabolism, valine, glucagon signaling pathway, leucine and isoleucine degradation, diabetic cardiomyopathy, glyoxylate and dicarboxylate metabolism, and central carbon metabolism in cancer (**Figure 7**).

### 3.5 Therapeutic agents screening for cuproptosis-related key genes

Therapeutic agents for the cuproptosis-related genes were screened using Enrichr. And the predicted results suggested that latamoxef, vitinoin, clomipramine, chlorzoxazone, glibenclamide, pyruvic acid, clindamycin, medrysone, caspan, and flavin adenine dinucleotide might be the target agents for cuproptosis-related genes (**Table 4**).

**Table 4:** Therapeutic agents for cuproptosis-related genes.

Term	*p*-value	Combined Score	Genes
latamoxef	1.39E-04	173.328	PDHA1, FDX10, DBT, DLAT, LIAS
Vitinoin	1.42E-04	219.439	PDHA1, DBT, PDHB, DLD
clomipramine	7.92E-04	446.560	DBT, LIAS
chlorzoxazone	9.66E-04	101.323	PDHA1, FDX1, DLAT, DLD
glibenclamide	0.001	95.586	PDHA1, FDX1, DLAT, DLD
Pyruvic acid	0.002	270.716	LIAS, DLD
clindamycin	0.002	68.639	PDHA1, FDX1, DLAT, LIAS
medrysone	0.003	90.444	FDX1, DLAT, LIAS
Caspan	0.003	82.339	ATP7B, DBT, DLAT
flavin adenine dinucleotide	0.004	1549.437	DLD

### 3.6 Altered expression of cuproptosis-related genes in DSS-induced colitis

**Figure 8:**
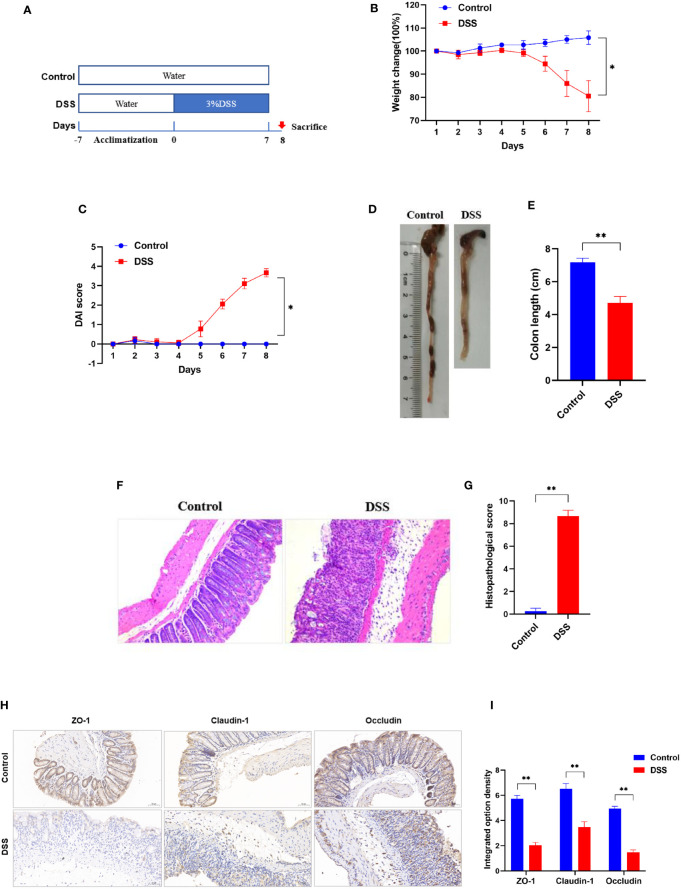
Successful establishment of DSS-induced colitis *in vivo*. **(A)** Schematic overview of DSS-induced colitis mice. **(B)** Daily changes in body weight and **(C)** DAI score in different groups (n = 6). **(D)** Representative colon pictures of mice from different groups and the comparison of colon length (n = 6). **(E)** Statistics of colon length. **(F)** Representative histopathological images of mice colon sections and histopathological scores (200× magnification, n = 6). **(G)** The histopathological score. **(H)** Representative images of immunohistochemical staining of ZO-1, claudin-1, and occludin in colonic tissues (200× magnification, n = 3). **(I)** The integrated option density was used in order to quantify ZO-1, claudin-1, and occludin proteins. *p < 0.05 compared to the control, **p < 0.005 compared to the control.

Compared to control group, the body weight and colon length of mice in DSS group were significantly decreased, while DAI score increased significantly (**Figures 8B–E**). In addition, disappearance of crypt glands, mucosal damage and inflammatory cell infiltration were observed in the colonic tissue of mice with colitis, while in the control group the colonic mucosa was intact, and the crypt structure was clear (**Figures 8F, G**). The IHC analysis results revealed that positive expression of ZO-1, claudin-1, and occludin were observed in the control group, however, they were significantly inhibited in the DSS group (**Figures 8H, I**). Taken together, these results indicate the successful establishment of an acute experimental colitis model.

**Figure 9:**
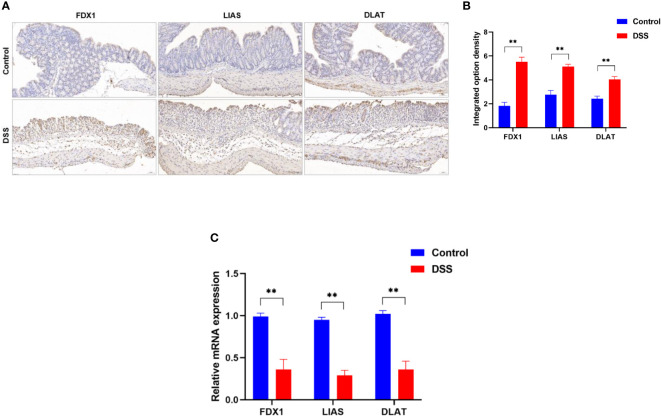
Altered expression of cuproptosis-related genes in DSS-induced colitis. **(A)** representative images of immunohistochemical staining of FDX1, LIAS, and DLAT in colonic tissues (200× magnification, n = 3). **(B)** The integrated option density was used in order to quantify FDX1, LIAS, and DLAT proteins. **(C)**FDX1, LIAS, and DLAT expression levels in colon tissues were detected by qRT-PCR (n = 6). **p < 0.005 compared to the control.

To further explore the role of cuproptosis in UC, genes that are essential for cuproptosis (6) were evaluated. The IHC analysis results revealed that positive expression of FDX1, LIAS and DLAT were observed in the control group, however, they were significantly inhibited in the DSS group (**Figures 9A, B**). Furthermore, the results of qRT-PCR (**Figure 9C**) revealed that the mRNA levels of FDX1, LIAS and DLAT were significantly altered in the DSS group compared to control group. Taken together, these results indicate that the cuproptosis-related genes presented well discrimination for UC, which validate the analysis results of microarray data.

## 4 Discussion

Copper metabolism plays an important role in gastrointestinal disorders. Excessive storage of copper may cause intestinal cell damage and even lead to pathological diseases. Cuproptosis is a novel kind of regulation of cell death that differs from other forms of regulation of cell death such as necroptosis, pyroptosis, and ferroptosis (**Table 5**) (6). In contrast to healthy individuals, UC patients showed copper accumulation (10–12). Regulation of cell death plays an important role in many pathological and physiological processes, including inflammatory bowel disease (24). To date, what role cuproptosis plays in UC has not been explored.

**Table 5:** Hallmarks of major types of regulation of cell death.

Type	Trigger factors	Morphological features	Representative inhibitors
Cuproptosis	Intracellular excess copper	Abnormal morphology of mitochondria	Tetrathiomolybdate
Alkaliptosis	Intracellular alkalinization	Necrosis-like morphology	N-acetyl alanine acid
Oxeiptosis	Accumulation of reactive oxygen species	Apoptosis-like morphology	N-Acetyl-L-cysteine
Autophagy-dependent cell death	Molecular machinery of autophagy	Autophagic vacuolization	Chloroquine
Ferroptosis	Intracellular iron accumulation	Smaller mitochondria; reduced mitochondria crista; elevated mitochondrial membrane densities	Ferrostatin-1
Parthanatos	Oxidative stress	Chromatin condensation; large DNA fragmentation	Iniparib
Entotic cell death	Activation of adhesion proteins and actomyosin	Cell-in-cell structure	C3-toxin
Immunogenic cell death	A relatively restricted set of stimuli	Not appliable	KIRA6
Necroptosis	Extracellular stimulation	Cell swelling; rupture of plasma membrane	Necrostatin-1
Netotic cell death	Formation of neutrophil extracellular traps	Plasma membrane rupture; nuclear membrane collapse; chromatin fibre release	Lactoferrin
Pyroptosis	Activation of inflammasome	Rupture of plasma membrane; bubbling	Ac-YVAD-cmk
Lysosome-dependent cell death	Release of lysosomal hydrolytic enzymes	Lysosome and plasma membrane rupture	CA-074Me
Apoptosis	Activation of caspases	Apoptotic body formation	Emricasan

UC is a chronic inflammatory bowel disease characterized by a dysregulated mucosal immune response. GSEA is a method to analyze gene sets in groups and can identify differential gene expression profiles among different phenotypes and groups. Whereas ssGSEA is an extension of GSEA to calculate separate enrichment scores for each pairing of a sample and gene set (25). In this way, ssGSEA converts the gene expression profiles of individual samples into gene set enrichment profiles. By defining immune cell and immune function-related gene sets, the enrichment score of the gene set can represent the immune cell and immune function characteristics of each sample (25). This transformation enables researchers to characterize the immune infiltrate profile of individual samples rather than by flow cytometry or immunohistochemistry (26). Developed and validated with microarray data, CIBERSORT is a method for characterizing the cellular composition of complex tissues from their gene expression profiles (27). CIBERSORT requires Gaussian distribution data, whereas unnormalized RNA-seq counts are negatively binomially distributed (27). Hence, RNA sequencing data must be converted to “microarray-like” data when analyzed before it can be used for subsequent analysis (28). Interestingly, ssGSEA does not require secondary transformation of the data when analyzing RNA sequencing data (26). In addition, ssGSEA is based on the actual analysis of immune cells and immune function-related genes and is not limited by immune cells and immune function types (25), whereas CIBERSORT can only evaluate a fixed set of 22 cell types (27). Therefore, the ssGSEA method was used to quantify the enrichment scores of immune cells and immune functions for each specimen in the present study.

We hypothesized that cuproptosis was involved in UC and that cuproptosis-related genes may contribute to the early diagnosis and treatment of UC. In the present study, microarray data was applied to reveal the potential significance of cuproptosis in the UC disease process. First, the immune infiltration characteristics of UC were identified, and the results revealed a significant difference in immune infiltration between the colonic tissue of UC patients and the normal group. This finding implies that there may be excessive survival and proliferation of multiple immune cells in UC patients, further mediating inflammation and barrier disruption in the intestine. Second, the association between cuproptosis-related genes and UC pathological states was explored, and the results suggested that cuproptosis-related genes were closely associated with immune infiltration in UC. We next identified key cuproptosis-related genes based on their degree of association with UC immune infiltration and used them to construct a risk prediction model for UC, which was found to have excellent discrimination. These results suggest that cuproptosis may be involved in UC, and the cuproptosis-related genes may serve as diagnostic biomarkers for UC. Furthermore, after successfully inducing experimental colitis in mice, genes that are essential for cuproptosis were evaluated using qRT-PCR and IHC, which revealed that the levels of FDX1, LIAS and DLAT in colonic tissues were significantly altered in the DSS group compared to control group, validating the analysis results of microarray data. These results all indicate that cuproptosis may be involved in UC and that its related genes presented well discrimination for UC.

The tricarboxylic acid cycle provides carbon for biosynthesis and a reducing agent for ATP production, which is essential for oxidative metabolism (29). Cuproptosis depends on copper binding to components of the tricarboxylic acid cycle and subsequent disruption of normal respiratory function of mitochondria (6). Thus, the tricarboxylic acid cycle is crucial for the regulation of cuproptosis. A markedly different profile of tricarboxylic acid cycle-related molecules has been found in colonic lesion tissue and serum of UC patients compared to healthy volunteers (30). D-2-hydroxyglutaric acid (D2HG) is a product of the tricarboxylic acid cycle, which a positive correlation was observed between the level of D2HG in the urine of colitis-associated colon cancer mice and the number of subsequent polyps and severity of dysplasia (31). Furthermore, intravenous administration of D2HG resulted in delayed recovery from colitis and severe tumorigenesis in mice (31). Therefore, excess copper might disrupt the tricarboxylic acid cycle and subsequent mitochondrial respiration causing death of intestinal epithelial cells, thereby disrupting the intestinal mucosal barrier, and ultimately inducing UC.

PDHB, PDHA1, LIAS, FDX1, DLD, DLAT, DBT, and ATP7B were included in the diagnostic model. The further bioinformatic analysis of these cuproptosis-related genes revealed the enrichment of some cell death, metabolic and immune response-related pathways. Pathways, such as tricarboxylic acid cycle, Pyruvate metabolism, Valine, leucine and isoleucine degradation, and HIF-1 have been identified to be involved in UC (32–34). Hence, cuproptosis-related genes may regulate the pathological process of UC by mediating these classical pathways associated with cell death, metabolism, and immune response.

As a novel mode of copper-induced cell death, therapeutic agents targeting cuproptosis are a gaping field. Therefore, therapeutic agents for the cuproptosis-related genes were screened in the present study. The predicted results suggested that latamoxef, vitinoin, clomipramine, chlorzoxazone, glibenclamide, pyruvic acid, clindamycin, medrysone, caspan, and flavin adenine dinucleotide might be the target agents for cuproptosis-related genes. These therapeutic agents, such as latamoxef and clindamycin belong to antibiotics family. It is well known that microbial dysregulation has been increasingly appreciated in the pathogenesis of inflammatory bowel diseases (35, 36), and antibiotics used to modulate gut microbes and fecal microbiota transplantation have been considered to treat UC (37). However, it remains to be verified whether antibiotics or fecal microbiota transplantation can target cuproptosis-related genes.

To our knowledge, this is the first study to explore the role of cuproptosis in UC. Although encouraging results were found, limitations should be acknowledged. First, the clinical data for this study were obtained from public databases, and the clinical information of the samples was incomplete, such as the clinicopathological characteristics of the GSE series were not available. Second, the data used in this study was constructed on RNA sequences. Although animal experiments validated the results of bioinformatics analysis, their reproducibility and broad applicability still need to be validated with clinical samples in the future, as we were unable to obtain enough clinical samples of UC within a tight timeframe. Third, results of IHC suggested that the protein expression level of cuproptosis-related genes was significantly increased in DSS-induced colitis mice, while the mRNA expression level of cuproptosis-related genes was significantly decreased in DSS-induced colitis mice, suggesting a complex regulatory mechanism of cuproptosis in UC. In this study, we could not explore the regulatory mechanism of copper death in UC within a tight timeframe, but further studies in the future are highly warranted. Fourth, almost all cuproptosis-related genes were significantly correlated with immune infiltration in UC, and these genes showed an excellent discrimination of UC, suggesting that cuproptosis may be a potential therapeutic target in UC and the predictive value of cuproptosis-related genes in the early diagnosis of UC. Admittedly, these findings provide new clues to investigate the role of cuproptosis in UC, however, more studies are still needed to confirm our findings.

## 5 Conclusion

In conclusion, our study revealed that cuproptosis was significantly associated with immune infiltration in UC, and cuproptosis-related genes showed an excellent discrimination for UC. Therefore, cuproptosis may be a therapeutic target for UC, and the model based on cuproptosis-related genes facilitates the early diagnosis of UC.

## Data availability statement

The original contributions presented in the study are included in the article/supplementary material. Further inquiries can be directed to the corresponding author.

## Ethics statement

The studies involving animals were reviewed and approved by the Animal Ethics Committee of Xi Yuan Hospital of China Academy of Chinese Medical Sciences (Approval NO. 2019XLC003-2).

## Author contributions

JH and JZ contributed equally to this paper. JH drafted the manuscript. JZ, FW, and BZ helped with implementation of this work. XT contributed to the methodology, review, and editing of the manuscript. All authors read and approved the final manuscript.

## Funding

This work was supported by the National Natural Science Foundation of China (No. 81830118), China Academy of Chinese Medical Sciences Innovation Fund (No. CI 2021A01012), China Academy of Chinese Medical Sciences Excellent Young Talent Cultivation Fund (No. ZZ 15-YQ-002), and Administration of Traditional Chinese Medicine Digestive Refractory Disease Inheritance and Innovation Team Project (No. ZYYCXTD-C-C202010).

## Conflict of interest

The authors declare that the research was conducted in the absence of any commercial or financial relationships that could be construed as a potential conflict of interest.

## Publisher’s note

All claims expressed in this article are solely those of the authors and do not necessarily represent those of their affiliated organizations, or those of the publisher, the editors and the reviewers. Any product that may be evaluated in this article, or claim that may be made by its manufacturer, is not guaranteed or endorsed by the publisher.
